# Simultaneous Determination of Furanic Compounds and Acrylamide in Insect-Based Foods by HPLC-QqQ-MS/MS Employing a Functionalized Mesostructured Silica as Sorbent in Solid-Phase Extraction

**DOI:** 10.3390/foods10071557

**Published:** 2021-07-05

**Authors:** Lorena González-Gómez, Sonia Morante-Zarcero, Damián Pérez-Quintanilla, Isabel Sierra

**Affiliations:** Departamento de Tecnología Química y Ambiental, E.S.C.E.T, Universidad Rey Juan Carlos, C/Tulipán s/n, Móstoles, 28933 Madrid, Spain; lorena.gonzalez@urjc.es (L.G.-G.); sonia.morante@urjc.es (S.M.-Z.); damian.perez@urjc.es (D.P.-Q.)

**Keywords:** acrylamide, 5-hydroxymethylfurfural, furfural, 5-methylfurfural, insect-based foods

## Abstract

Insect-based products are novel foods (NF) that merit careful study. For this reason, in this work a method has been developed for the simultaneous analysis of four food processing contaminants (FPC), acrylamide (AA), 5-hydroxymethylfurfural, (HMF), 5-methylfurfural (MF) and furfural (F), in insect-based products (bars, crackers and flours) by high-performance liquid chromatography coupled to triple quadrupole mass spectrometry (HPLC-QqQ-MS/MS). The method consisted of a solid-liquid extraction (SLE) with acidified water, followed by solid-phase extraction (SPE), using 100 mg of a sorbent based on mesostructured silica with a large pore functionalized with amino groups (SBA-15-LP-NH_2_). The analytical method was properly optimized and validated in a representative bar sample of pineapple & coconut with cricket flour (Ins-B-Pine-Coco) showing good accuracy, with recoveries ranging from 70–101% for the four analytes and adequate precision (RSD < 9%). Good linearity (R^2^ ≥ 0.995) and low method quantification limits for AA (between 1.3–1.4 µg/g), F (between 7.9–8.8 µg/g), MF (between 3.1–6.5 µg/g) and HMF (between 1.5–3.3 µg/g) were also obtained in all samples studied. The proposed method was successfully applied in eleven insect-based foods. Results revealed that insect-based bars can be a good alternative to traditional cereal bars to reduce dietary exposure to HMF; but, in order to reduce the exposure to AA, alternative formulations must be evaluated in the design of innovative insect-based crackers.

## 1. Introduction

Nowadays, there is a growing interest in the development of novel foods (NF), i.e., innovative foods produced with new raw materials or new production processes. Insect-based foods are being evaluated as NF, as they can be considered as a more sustainable alternative for the environment [1,2,3,4,5]. The production of edible insects generates a lower impact on the environment than conventional livestock, since less consumption of feed, water and land is needed for their rearing [4]. Moreover, fewer greenhouse gases are generated in their production [2,4]. Edible insects contribute to the diet by providing a variety of macronutrients and micronutrients, especially a high content of proteins, monounsaturated and polyunsaturated fatty acids [3,6,7]. Although entomophagy is common in many cultures, in most European countries it meets rejection [4]. Nonetheless, the Regulation (EU) 2015/2283 of the European Parliament includes insects as NF, and since 2018 this is being applied in all the member states of the European Union [8]. To avoid consumer rejection of insects, different types of insect-based products have emerged on the market. Some of these products (cookies, crackers, bars, pasta, etc.) are being offered as an alternative for people with celiac disease, since insect flours are gluten free. In addition, these products are also offered for athletes due to their high protein content. Population surveys have revealed that young people show interest in these types of products [3,9]. Therefore, this may lead to a change in consumption habits, including these products in the diet.

Despite the fact that these types of NF have many advantages, they should be carefully studied. In 2015, the European Food Safety Authority (EFSA) evaluated the biological and chemical risk of the production and consumption of insects as food or feed and demonstrated that the intake of these products can cause an allergic reaction in those people allergic to shellfish, crustaceans or mites [10,11]. Nevertheless, other risks still need to be assessed. For example, food processing contaminants (FPC), such as acrylamide (AA) and furanic aldehydes, among others, are causing great concern due to their toxicity. These FPCs are part of the well-known Maillard reaction (MR) or browning reaction. Particularly, AA is a toxic organic compound formed mainly by the reaction of asparagine with reducing sugars, which is very common in foods rich in starch subjected to high processing temperatures (generally above 120 °C) and with low moisture [12]. In 1994, AA was classified as a probable carcinogen (group 2A), according to the International Agency for Research on Cancer (IARC) [12,13,14]. On the other hand, 5-hydroxymethylfurfural (HMF), 5-methylfurfural (MF) and furfural (F) are intermediate products generated in MR and in the caramelization reaction [15,16,17,18]. Currently, there are few studies of the formation of FPC in insect-based foods. In a recent study by González et al. [19], a high concentration of HMF was found in an insect bar. On the other hand, in a new EFSA scientific opinion on the safety of *Tenebrio molitor* larva, results regarding the formation of AA in two bakery products made with mealworm were not conclusive [20]. Therefore, it is necessary to broaden the data collection in this field.

Up to date, AA and furanic compounds have been analysed in conventional foods, such as bread [14], breakfast cereals [15], coffee [21], snacks [22,23], Chinese commercial foods [24], caramel colors and beverages [25] or dairy formulas [26], using high-performance liquid chromatography (HPLC) with diode array detector [14] or coupled to mass spectrometry [19,23,25]. Nevertheless, no chromatographic method that performs the simultaneous analysis of AA, F, MF and HMF in foods has yet been described.

On the other hand, sample preparation is a very important stage in the analysis of FPCs, due to the complexity of the matrices where they are found. In the case of insect-based products, such as cookies or bars, besides insect flour they contain other ingredients, such as nuts, chocolate, cinnamon, oregano, thyme, honey, sugar, salt, etc. All these ingredients provide a large number of carbohydrates, fats and proteins, which together with the high number of proteins found in insects make the analysis of these foodstuffs very difficult. Usually, solid-liquid extraction (SLE) with polar organic solvents [23], acidified water [21,26,27] or mixtures of water with organic solvents [24] in combination with reagents to clarify and eliminate interferences (Carrez I and II) are used for the analysis of conventional foods. Nonetheless, to reduce matrix effects, to remove interferences and to improve the limits of the method, SLE has also been combined with solid-phase extraction (SPE) [14], dispersive solid-phase extraction (dSPE) [24] or the modified QuEChERS (Quick, Easy, Cheap, Effective, Rugged & Safe) protocol [25] using commercial sorbents. In this sense, the use of new materials as sorbents can offer interesting advantages over commercial ones, due to their good structural characteristics and their flexibility when functionalized with different ligands. At the moment, new materials have been used for the extraction of AA, such as molecular printing polymers [28,29], multiwalled carbon nanotubes [30], magnetic nanomaterials [31,32] or silica nanoparticles [29]. Moreover, mesostructured silica-based materials are a current trend in the extraction of toxic compounds [33,34,35] and their application to the determination of HMF in cereal and insect bars has been demonstrated recently [19]. 

In this work, a mesostructured silica with a large pore functionalized with aminopropyl groups (SBA-15-LP-NH_2_) has been evaluated as sorbent in SPE for the extraction of AA, F, MF and HMF. A new chromatographic method for the simultaneous analysis of these four PFCs by HPLC-QqQ-MS/MS has been developed and successfully applied to their determination in 11 insect-based food samples.

## 2. Materials and Methods

### 2.1. Reagents and Materials

AA (≥99%, CAS 79-06-1), F (≥98.5%, CAS 98-01-1), MF (≥98.5%, CAS 620-02-0) and HMF (≥98%, CAS 67-47-0) was purchased from Sigma–Aldrich (St. Louis, MO, USA). Stock standard solutions (1000 mg/L) were prepared by diluting 10 mg of each analyte in 10 mL of methanol (MeOH) and stored at −20 °C in darkness. Working standard solutions (0.05–250 mg/L) were prepared by appropriate dilution of the stock solutions with water-MeOH (90:10, *v/v*) and were stored at 2–8 °C. LC-MS grade MeOH, acetonitrile (ACN) and hexane (Hex) were obtained from Mervilab (Madrid, Spain). Formic acid LC-MS grade was acquired from Thermo Fisher Scientific (Leicestershire, UK). Hydrochloric acid (HCl, 37%, 36.45 g/mol, 1.19 g/mL), toluene, ethanol and diethyl ether were purchased from Scharlau (Barcelona, Spain). Tetra-ethyl-orthosilicate (TEOS, 98%, 208.3 g/mol, 0.93 g/mL), poly (ethylene-glycol)—block-poly (propylene-glycol)—block-poly (ethylene-glycol) (PEG-PPG-PEG or Pluronic 123^®^, 5800 g/mol, 1.019 g/mL), 3-aminopropyl-triethoxysilane (≥98%, 221.37 g/mol), decane (≥95%), potassium hexacyanoferrate and zinc sulfate analytical grade were obtained from Sigma-Aldrich (St. Louis, MO, USA). Ammonium fluoride (NH_4_F) was acquired from Panreac (Castellar del Vallès, Barcelona, Spain). All water used in this work was ultra-pure water (18.2 MΩ cm quality) obtained from a Millipore Milli-Q-System (Billerica, MA, USA). Polyethylene frits, nylon filter membranes (0.45 µm), empty SPE cartridges (3 mL) and nylon syringe filters (0.45 µm) were purchased from Scharlau (Barcelona, Spain). Carrez solutions used during sample preparation were prepared by dissolving 1.5 g of potassium hexacyanoferrate in 10 mL of water (Carrez I) and 3 g of zinc sulfate in 10 mL of water (Carrez II).

### 2.2. Samples 

Eleven insect-based products were purchased from different local markets in Madrid (Spain). Insect-based food samples analyzed were: a cricket flour (100%, *Acheta domesticus*), a cricket flour with chocolate, six cricket bars and three cricket crackers. Three cereal bars were also purchased and analyzed for comparative purposes.

Before extraction, samples were milled and homogenized using a mincer (A11 Basic analytical mill, IKA, Staufen, Germany). Afterwards, the samples were sieved to achieve a homogeneous particle size. Finally, the samples were stored at room temperature and were codified indicating whether they were insect (Ins) or cereal (Cer), followed by a capital letter that indicates the type of product: bar (B), cracker (C) or flour (F), and the initials of the most characteristic ingredient of the product (see Table S1 in supplementary material). The ingredients declared on the label of each product and the nutritional facts for each sample are shown in Tables S1 and S2.

### 2.3. Synthesis of Functionalized Mesostructured Silica

SBA-15-PE-LP functionalized with aminopropyl groups (SBA-15-LP-NH_2_) was synthesized according to [19]. First, SBA-15-LP type silica was synthesized by dissolving 12 g of Pluronic 123^®^ (Aldrich, St. Louis, MO, USA) in 420 mL of 0.1% HCl (*v/v*) and stirred in a silicone bath at 30 °C. When the Pluronic 123^®^ was completely dissolved, 0.14 g of NH_4_F was added and stirred for 10 min (450 rpm). Later, 25.84 g of TEOS and 75 mL of decane were added at the same time drop by drop. Next, the mixture was left for 20 h with a stirring of 300 rpm and at a temperature of 30 °C. After this time, the mixture was transferred into a Teflon-lined stainless steel autoclave at 100 °C for 48 h. The mixture obtained was filtered under vacuum, washed with Milli-Q water, air-dried, ground and calcined. The calcined program was from 20 to 540 °C at 1 °C/min, 8 h at 540 °C). Once the silica was calcined, it was functionalized with aminopropyl groups (SBA-15-LP-NH_2_). The synthesis was prepared in 1:1 ratio (*w/v*); for this, 2.2 g of SBA-15-LP was mixed with 40 mL of toluene and 2.2 mL of 3-aminopropyl-triethoxysilane (NH_2_) was added. The mixture was heated at 80 °C and stirred for 24 h at 400 rpm under an inert atmosphere. Later, the material obtained, named SBA-15-LP-NH_2,_ was filtered and washed with 40 mL of different dried solvents (toluene, ethanol and ethyl ether) and dried at vacuum overnight at room temperature.

### 2.4. Characterization of Mesostructured Silicas

The synthesized material was characterized by nitrogen gas adsorption-desorption isotherms, elemental analysis (EA) and scanning electron microscopy (SEM). Nitrogen gas adsorption-desorption isotherms were obtained using a Micrometrics analyzer (ASAP 2020, Micromeritics, Norcross, Georgia, USA). The Brunauer–Emmett–Teller (BET) method was used to calculate the surface specific area (S_BET_) and the Baret–Joyner–Halenda (BJH) model of the desorption branch was used to obtain the pore size distribution. For this analysis, 0.2 g of material was dried under vacuum overnight. EA was carried using a Flash 2000 Thermo Fisher Scientific Inc. SEM images were obtained through a Nova NanoSEM 230 FEI. For this analysis, the material was previously dispersed in ethanol and covered with a thin layer of gold.

### 2.5. Sample Preparation

**Figure 1:**
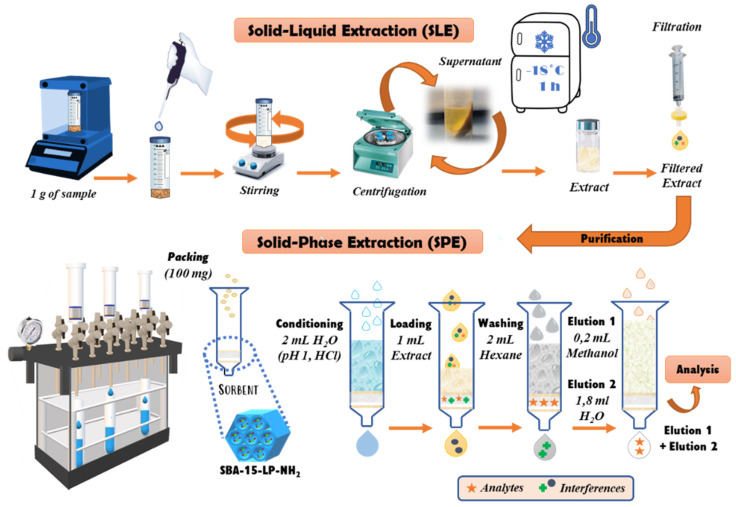
Schematic diagram of sample preparation: solid-liquid extraction (SLE) step and solid-phase extraction (SPE) step.

1 g of sample (±0.0001 g) was weighed, and it was mixed with 10 mL of cold water acidified with HCl (pH 1.0). The mixture was stirred for 15 min at room temperature. Later, 0.1 mL of Carrez I and 0.1 mL of Carrez II were added to the mixture, the sample was shaken for 5 min and then it was centrifuged at 6000 rpm for 10 min using a Rotofix 32A centrifuge (Hettich, Tuttlingen, Germany). The supernatant was separated from the precipitate and frozen for one hour. After this time, the frozen supernatant was again centrifuged at 6000 rpm for 10 min and filtered through a 0.45 µm nylon filter. Finally, 1 mL of sample extract was purified by SPE (Figure 1). 

To carry out the SPE procedure, 100 mg of the SBA-15-LP-NH_2_ was packed in empty SPE cartridges of 3 mL capacity. For this, the material was plugged with frits of polyethylene at both ends and a nylon membrane (0.45 µm) on the lower frit was used to avoid the loss of material. After the cartridges were packed, the SPE procedure was carried out using a Supelco Visiprep SPE vacuum manifold 12 port model (Sigma Aldrich, St. Louis, MO, USA) connected to a vacuum pump at 7.6 psi. In the first place, 2 mL of acidified water (pH 1.0, HCl) conditioned the cartridge and later, 1 mL of sample extract was loaded and passed through the cartridge. Afterwards, the cartridge was washed with 2 mL of Hex to eliminate interferences and, finally, the analytes were eluted with 0.2 mL of MeOH and later with 1.8 mL of water (Figure 1). The extract was filtered through a 0.45 µm nylon filter and it was injected into the HPLC-QqQ-MS/MS. All solvents used in the SPE procedure were previously cooled in a fridge at a temperature of between 0–5 °C.

### 2.6. HPLC-QqQ-MS/MS Conditions

AA, HMF, MF and F analysis was carried out with a Varian equipment and a data acquisition system MS Workstation version 6.3 (1200/1200 LC-MS/MS, Varian, Ibérica, Madrid, Spain). This HPLC contained two solvent delivery modules ProStar 210/215, a ProStar 410 autosampler (100 μL loop), a thermostatic column compartment. On the other hand, the HPLC was coupled to a triple quadrupole mass spectrometer (1200 L TQ) with electrospray ionization (ESI) ion source. The analytical separation was performed using a C18 Kromaphase 100 column (150 mm × 2.0 mm I.D., 3.5 μm particle size) with a C18 Kromaphase guard column (10 mm × 4.0 mm I.D., 5 μm particle size) at 30 °C and two mobile phases: solvent A (ACN) and solvent B (Milli-Q Water), both phases containing 0.1% formic acid, in gradient elution mode. The gradient elution started at 10% A and 90% B, then eluent A increased linearly to 90% in 9 min, remained in isocratic until minute 11 and returned to the initial conditions in 2 min, and was kept under these conditions for a further 2 min, at a constant flow rate of 0.3 mL/min. The total run-time of the method was 15 min and the injection volume was 10 µL. 

Mass spectrometry acquisition was carried out using an ESI ion source in positive mode for all analytes. N_2_ was used as the nebulizing and drying gas in the following conditions: drying gas N_2_ (350 °C and 22 psi), nebulizer gas pressure (60 psi), ion spray voltage (−5000 V for capillary and −600 V for shield). Argon was used as the collision gas at 1.90 mTorr and the detector was set at 1480 V. The ionization source parameters were optimized by direct infusion of a standard solution of AA, F, MF and HMF in MeOH (10 mg/L) at a flow rate of 20 µL/min. For the detection of all analytes, a multiple reaction monitoring (MRM) mode was used (mass peak width Q1 2.5; mass peak width Q3 2.5; scan width in MRM 0.70) and compounds were monitored at cone voltage (40 V). Table 1 shows mass spectrum parameters, product ions and ions used for quantification.

### 2.7. Instrumental Validation

To evaluate the instrumental parameters such as linearity, precision or detection (LOD) and quantification (LOQ) limits, different standard solutions were prepared and analysed on the HPLC-QqQ-MS/MS. Linearity was evaluated by analyzing standard solutions between 0.05–10 mg/L in water-MeOH (90:10, *v/v*). Repeatability and reproducibility were evaluated at two concentrations levels, low (0.5 mg/L) and high (2.5 mg/L). Repeatability was obtained by analyzing the standard solution six times in one day (*n* = 6) and reproducibility was evaluated by analyzing the standard solution three times, on three different days (*n* = 9). LOD and LOQ were calculated as three times and ten times the signal-to-noise ratio (S/N), respectively, obtained after analyzing the standard solution with the lowest concentration, 0.05 mg/L for AA, MF and HMF and 0.5 mg/L for F.

### 2.8. Method Validation

The optimized method was validated in terms of linearity, matrix effect (ME), precision, accuracy, method detection (MDL) and quantification (MQL) limits following previous works of this group [19,33] and according to the recommendations [36]. A pineapple and coconut insect bar (Ins-B-Pine-Coco) sample was used as a representative sample to carry out the complete validation. Linearity was evaluated with a matrix-matched calibration curve. To do this, after applying the optimized sample preparation protocol, an aliquot of a standard solution containing AA, F, MF and HMF was added to the extract to achieve the desired concentration level of the calibration curve. In turn, a blank sample (unspiked) was prepared for the signal correction in case the sample was contaminated. Linear regression analysis was applied. For this, seven known concentration levels were used, within the linear range evaluated for each analyte, to prepare the calibration curves by plotting the analyte concentration versus the peak area of each analyte. Linearity was expressed as the coefficient of determination (R^2^). The ME was calculated by comparing the slope of solvent-based standard calibration with the slope of the matrix-matched calibration and applying the following formula: ((slope matrix-matched/slope solvent-based − 1) × 100). Precision and accuracy were evaluated by doping the Ins-B-Pine-Coco sample at the beginning of the process and comparing it with a sample doped at the end of the process, at two different levels, corresponding to the legislated level in honey for HMF (high) [37] and the MQL level (low). Precision expressed as relative standard deviation, (% RSD) was evaluated in terms of repeatability (intra-day precision), six replicates in one day (*n* = 6) and reproducibility (inter-day precision), three replicates in three different days (*n* = 9). Accuracy expressed as recovery percentage (%) was evaluated through six replicates (*n* = 6), comparing the areas of the doped samples at the beginning of the process with the areas of the simulated samples at the end of the process. Finally, MDL was estimated as three times and MQL as ten times the S/N ratio for the chromatographic response obtained in HPLC-QqQ-MS/MS at the lowest concentration in the matrix-matched calibration.

To quantify analytes in all the insect-based samples, matrix-matched calibration was also carried out for a representative sample of crackers (Ins-C-Toma) and flours (Ins-F) and the ME, MDL and MQL were calculated.

## 3. Results and Discussion

### 3.1. Characterization of Mesostructured Silicas

**Figure 2:**
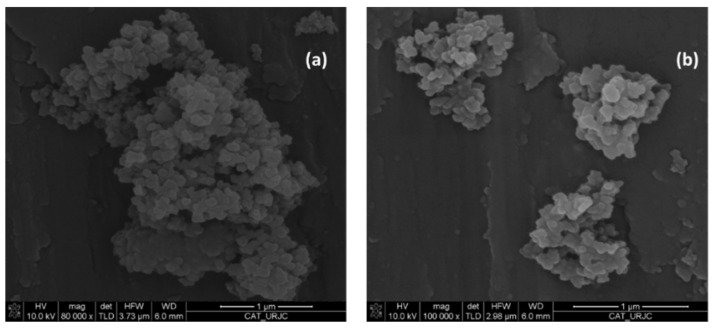
SEM images of SBA-15-LP-NH_2_ (**a**) 80,000× (**b**) 100,000×.

The N_2_ adsorption-desorption isotherms for the synthesized materials were type IV with a H1 hysteresis loop according to the International Union of Pure and Applied Chemistry (IUPAC) classification. The non-functionalized material (SBA-15-LP) exhibits a high S_BET_ (482 m^2^/g), pore volume (1.7 cm^2^/g) and pore diameters (91 and 38 Å) typical of mesostructured materials (Table 2). After functionalization, SBA-15-LP-NH_2_ presented a decrease in S_BET_ from 482 to 357 m^2^/g, pore volume from 1.7 to 1.3 cm^2^/g and pore diameter from 38 to 36 Å and 91 to 89 Å. EA revealed a degree of functionalization of 0.96 mmol of NH_2_ groups per g, according to what was previously published by the group [19]. SEM images were used to determine the morphology of the functionalized mesostructured silica (SBA-15-LP-NH_2_). Figure 2a (80,000×) and Figure 2b (80,000×) show that the material has a spherical or quasi-spherical form.

### 3.2. Optimization of HPLC-QqQ-MS/MS Conditions

In order to achieve an adequate separation of AA, F, MF and HMF, different mobile phases and gradients have been compared. So far, no chromatographic method that performs simultaneous analysis of AA, F, MF and HMF in foods been described.

**Figure 3:**
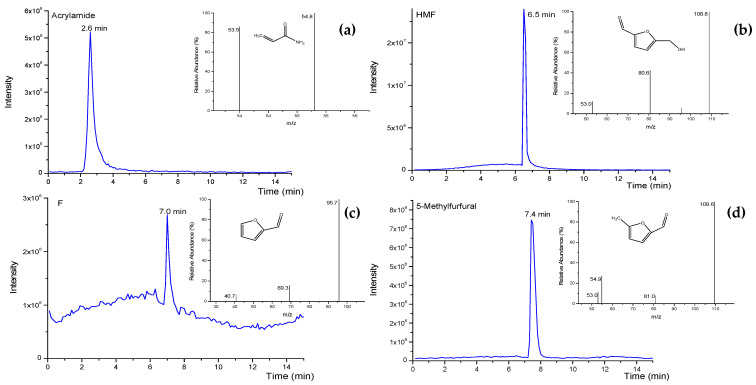
Extracted ion chromatograms obtained in a standard solution mixture of acrylamide (AA), furfural (F), 5-methylfurfural (MF) and hydroxymethylfurfural (HMF) (5 mg/L) in water-MeOH (90:10, *v/v*) with optimal gradient. (**a**) Extracted ion chromatogram (*m*/*z* 72.0 > 54.8) and mass spectrum for AA, (**b**) extracted ion chromatogram (*m*/*z* 127.0 > 108.8) and mass spectrum for HMF, (**c**) extracted ion chromatogram (*m*/*z* 97.0 > 95.7) and mass spectrum for F and (**d**) extracted ion chromatogram (*m*/*z* 110.9 > 109.6) and mass spectrum for MF.

Firstly, the chromatographic conditions optimized in a previous work of the group for HMF analysis were taken as a reference [19]. Gradient elution was performed by combining MeOH (eluent A) and Milli-Q water (eluent B), both containing 0.1% formic acid and 2 mM ammonium acetate. The gradient elution started at 10% A and 90% B, then eluent A increased linearly to 90% in 10 min and returned to the initial conditions in 5 min. Results showed good separation for the compounds (AA: 1.9 min, F: 4.6 min, HMF: 5 min and MF: 6.5 min), but wide peaks for AA, F and MF and low retention time for AA was observed. Under similar conditions (but without the addition of ammonium acetate to the mobile phase) ACN was tested as an organic phase, according to other works [23,24]. In these conditions, peaks were narrower and with better peak intensity, except for AA (wide peak at 2 min). Therefore, using ACN as eluent A, different gradients were evaluated, starting with 10 or 30% of ACN and increasing linearly to 60% or 90% in 5–10 min (flow rate of 0.3 mL/min) to optimize separation. Under the best conditions (from 10% to 90% ACN in 9 min) the retention time was: 2.6 min for AA (Figure 3a), 6.5 min for HMF (Figure 3b), 7.0 min for F (Figure 3c) and 7.4 min for MF (Figure 3d). Figure 3 shows the extracted ion chromatogram and the mass spectrum obtained for each analyte. This gradient was selected, even though AA showed poor retention. This is due to its high polarity which makes it difficult to achieve higher retention time with non-polar columns such as the C18 [38,39].

### 3.3. Optimization of Sample Extraction Procedure

#### 3.3.1. Preliminary Studies

The goal of this work was to develop an analytical method for the simultaneous determination of AA, F, MF and HMF in insect-based foods. For this, SBA-15-LP-NH_2_ was used, as it was previously tested with good results as SPE sorbent for HMF analysis [19]. The sample extraction procedure consisted of a SLE, with acidified water, followed by SPE with 100 mg of SBA-15-LP-NH_2_. In a preliminary study, sample extraction was carried out according to [19] using an Ins-B-Pine-Coco sample, doped with 40 µg/g of each analyte at the beginning (doped sample) and at the end (simulated sample) of the extraction process. Results showed recoveries close to 100% for HMF, but around 20% for AA. No peaks were observed for MF and F, neither in doped samples nor in simulated samples, so it was supposed that loss of both analytes was due to evaporation. Consequently, assays were repeated changing the vortex agitation by plate shaking (15 min) and avoiding the evaporation stage. In addition, during the extraction and purification process, cold solvents were used, stored until their use in a refrigerator at 5 °C. In these conditions, recovery percentages increased to 40% for AA and MF and 60% for F. It is well known that MF and F are volatile organic compounds, and for this reason, many sample preparation protocols are only based on SLE, without a purification step [16,21,27,40]. However, these extraction protocols cannot reduce the ME, making it difficult to identify and analyze the target compounds, negatively affecting the useful life of the chromatographic column. For this reason, SPE is necessary to purify the extract and reduce the interferences caused by the components of the sample in the MS detection.

#### 3.3.2. SPE Optimization

Firstly, the capacity of the SBA-15-LP-NH_2_ for the retention of the four analytes was evaluated. For this purpose, two assays were carried out with standard solutions at different concentration levels and with different amounts of sorbent. First, different amounts of SBA-15-LP-NH_2_ (50, 100 and 150 mg) were studied at a high concentration level (80 µg/mL) to determine if the active sites of the material were enough. The cartridge was conditioned with 2 mL of acidified water (pH 1.0, HCl), and then 1 mL of the standard solution (in acidified water) was loaded. The cartridge was washed with 2 mL of Hex and, finally, the analytes were eluted with 3 mL of MeOH. Before injecting into the HPLC-QqQ-MS/MS, the eluate was diluted with 3 mL of ACN to avoid the splitting of the AA peak (which was observed when this analyte was injected in MeOH). Results in Figure S1a (supplementary material, SM) showed that better recoveries were found as the amount of sorbent was increased. This phenomenon can be attributed to the higher number of active sites available in the amount of sorbent to interact with the target analytes. A second assay was carried out using standard solutions at low concentration levels (10 and 5 µg/mL), which are close to those reported in other sample extracts, according to the literature [19,22]. For this study, 100 mg of material were used to verify if this amount of sorbent was enough for these concentration levels. The extraction conditions were the same as those described above for the first assay. The results showed good recovery percentages (70–113%) for the four analytes using 100 mg of sorbent (Figure S1b, SM). Therefore, this amount of sorbent was selected for the proposed analysis. However, for higher amounts of analyte, more sorbent material could also be used, if needed. 

Subsequently, the elution in the SPE was optimized using 100 mg of material in order to eliminate the dilution step of the eluate with ACN. For this, firstly a study of the effect of type solvent on the shape of the chromatographic peaks was carried out. Different solvents and solvent mixtures were tested: water, MeOH-ACN (50:50, *v/v*), water-MeOH (50:50, *v/v*), water-MeOH (70:30, *v/v*), water-MeOH (90:10, *v/v*). In the mixtures water-MeOH 50:50 and 70:30 (*v/v*) a splitting in AA peak was observed (as it was found with pure MeOH). In the other media, the peaks were narrow without splitting and with similar intensity; therefore, these media were tested for the elution step in the SPE. Thus, 1 mL of a standard solution (5 µg/mL) prepared in acidified water was loaded in cartridges previously conditioned, and after washing with Hex, the analytes were eluted with 3 mL of water, MeOH-ACN (50:50, *v/v*) and water-MeOH (90:10, *v/v*). On the other hand, a sequential elution was carried out as follows: 1st eluent: MeOH (0.3 or 0.6 mL) and 2nd eluent: water (2.7 or 2.4 mL). In Figure S2a (SM) the recoveries of AA, F, MF and HMF are shown. In this figure, results obtained are compared with the recovery percentage obtained using pure MeOH (but diluting to 50% with ACN before the injection). As can be seen, good recoveries, in general, higher than 70%, were found. Water appears to recover all analytes well, except HMF, so this solvent was discarded. By using MeOH-ACN (50:50, *v/v*) as eluent, recoveries of 66% were found for AA and with water-MeOH (90:10, *v/v*) recoveries of 70% were achieved for HMF. Additionally, it was observed that a sequential elution improved the recoveries of all the analytes. The first portion of MeOH followed by water seems to contribute to the weakening and breakdown of the ion-dipole forces between the amino groups and the silanol groups (Si–OH) of the silica surface (positively charged at pH 1 in the analyte adsorption process, lower than the Point of Zero Charge, PZC, of the surface) with the polar target analytes in favor of the hydrogen bonding between the solvent and the analytes. Finally, with the sequential elution, the solvent volume was reduced to 2 mL to increase the preconcentration. In Figure S2b high recoveries (78–111%) can be observed for the four analytes, even though it seems that a greater amount of water helps the elution of MF and F. For this reason, the optimized conditions for the elution step were first eluent MeOH (0.2 mL) and second eluent water (1.8 mL).

#### 3.3.3. Application of the Optimized SLE-SPE Protocol

**Figure 4:**
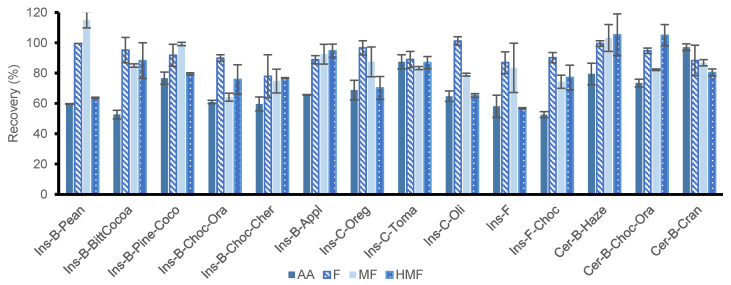
Recovery percentage (%) of acrylamide (AA), furfural (F), 5-methylfurfural (MF) and hydroxy-methyl-furfural (HMF) obtained in the study with samples doped at high level (50 µg/g) with optimized conditions of SLE-SPE procedure.

The optimized SLE-SPE protocol was tested in the 11 insect-based foods. 1 g of sample was doped with 50 µg/g (equivalent to 5 µg/mL in the extract) of the target analytes, 10 mL of acidified water (pH 1.0, HCl) were added and the sample was stirred for 15 min on a plate. After this time, the Carrez I and II solutions were added and the sample was stirred for 5 min. Subsequently, it was centrifuged and the supernatant was frozen for 1 h, to help the precipitation of the interferences. Then it was centrifuged again and 1 mL was filtered and loaded into the SPE cartridge. After the cartridge was washed, the analytes were eluted with MeOH (0.2 mL) + water (1.8 mL). Figure 4 shows the results obtained. Recoveries were between 53–87% for AA, between 78–101% for F, between 64–115% for MF and between 57–95% for HMF. In some samples, low recoveries were found for some analytes, especially in the case of AA. This fact may be due to the complexity of these matrices, as insect bars, crackers and flours also contain many other ingredients, such as nuts, chocolate, herbs and spices, honey, sugar, salt, etc. All these ingredients provide a large number of compounds that hinder the interactions produced between the analytes and the material and, therefore, affects the analyte recovery. Additionally, and for comparative purposes, three samples of cereal bars (hazelnut, chocolate and orange and cranberry bars) were analyzed with the proposed SLE-SPE protocol, and in these samples, in general, better recoveries were found (between 73–97%, 88–100%, 82–103% and 80–105% for AA, F, MF and HMF, respectively). This highlights the complexity of these samples.

### 3.4. Instrumental Validation

For the instrumental validation, standard solutions were prepared in water-MeOH (90:10, *v/v*). This medium was selected because it was the best elution medium after SPE optimization (see Section 3.3.2). Linear range, LOD, LOQ, repeatability and reproducibility were evaluated. The linear range was 0.05–10 µg/mL for AA and HMF, 0.5–10 µg/mL for MF and 1–10 µg/mL for F with R^2^ > 0.996 in all cases (Table S3, supplementary material). Low LOD and LOQ were obtained (Table S3): 0.03 and 0.10 µg/mL for AA, 0.22 and 0.72 µg/mL for F, 0.05 and 0.17 for MF and 0.01 and 0.03 for HMF, respectively. Repeatability and reproducibility were less than 8% and 9%, respectively, for all analytes.

### 3.5. Method Validation

Table 3 shows the linear range, matrix-matched calibration curve, MDL, MQL and ME for AA, F, MF and HMF in a representative sample of insect bars (Ins-B-Pine-Coco), insect cracker (Ins-C-Toma) and insect flour (Ins-F) applying the proposed method. The matrix-matched calibration curves showed a good linear regression with R^2^ ≥ 0.995 in all the studied samples (Table 3). Regarding the ME, an important increase in the signal for AA was observed. On the contrary, a significant matrix suppression of around—65% for HMF was observed in Ins-F and Ins-C-Toma. In contrast, almost no ME was found for the target analytes in the other samples analyzed. Therefore, to quantify simultaneously AA, F, MF and HMF in this kind of samples, matrix-matched calibration curves should be used to compensate errors associated with ME and in order to perform a true quantification of the four compounds. 

As can be seen in Table 3, in the cricket flour sample (Ins-F), none of these parameters could be calculated for AA, due to an interference that avoids the correct integration of the peak area of this compound (Figure S3). In some papers, this interference has been identified as the essential amino acid valine [39], because this amino acid has the same product ion as AA. Thus, it is expected to find this interference in this sample because Ins-F is composed of 100% house cricket (*Acheta domesticus*). This insect contains around 61% proteins, and valine is found to be in amounts between 48–60 mg/g of protein [6]. In the other insect-based samples, the cricket flour is found in amounts between 8%–20% of the product (Table S1), so this amino acid appears as a small peak in front of the AA, without affecting its quantification.

The limits of the method were calculated for the three samples evaluated (Table 3). For AA, MQL between 1.3–1.4 µg/g, for F, MQL between 7.9–8.8 µg/g, for MF, between 3.1–6.5 µg/g and for HMF between 1.5–3.3 µg/g were estimated. It deserves to be mentioned that none of these analytes has legislation for this type of sample. In the case of AA, Regulation 2017/2158 [41] establishes reference levels between 0.05 and 4 µg/g in some other foods to which the regulation refers. 

Finally, the precision and accuracy were evaluated in the Ins-B-Pine-Coco sample (Table 4). Precision showed satisfactory results for both intra-day and inter-day precision, showing RSD (%) ≤ 11% for AA and F, ≤8% for MF and ≤5% for HMF in the case of intra-day precision and RSD (%) ≤ 11% for AA, % RSD ≤ 14% for F, equal to 11% for MF and ≤8% for HMF in the case of inter-day precision. Accuracy showed recoveries ranging from 70%–101% for the four analytes (Table 4).

### 3.6. Analysis of Real Samples

The optimized method was applied to the analysis of 11 insect-based foods (Table 5). The products based on cricket (*Acheta domesticus*) were six bars, three crackers and two flours, with different ingredients. The ingredient list and the nutritional information for each product can be found in Tables S1 and S2, respectively (supplementary material). Each sample was analyzed in triplicate with the SLE-SPE-HPLC-QqQ-MS/MS method and each extract was injected three times. Ins-B-Pine-Coco, Ins-C-Toma and Ins-F were used as representative samples to prepare matrix-matched calibration curves for quantification of the target analytes in the whole collection of bars, crackers and flour samples. Additionally, for comparative purposes, three samples of cereals bars were analyzed with the proposed method. To date, there are no data on the presence of these FPC in this type of sample. Figure S3 (SM) shows the chromatogram of three representative contaminated samples. 

AA is an FPC formed when foods containing free asparagine and reducing sugars (both naturally occurring in cereals) are heated at temperatures higher than 120 °C (which can be reached by frying, baking and grilling). The amount of AA formed depends on the temperature, the time and the food composition/ingredients. AA can be found at different levels in foods made with cereals, such as snacks, bread and fine bakery wares (biscuits, crackers, crispbread), among others. As it was expected, AA was not found in insect flours, insect bars and cereals bars (Table 5), as these products are not heated at high temperatures during their manufacture and because AA is absent or present in a limited way among their ingredients [42,43,44]. On the other hand, AA was detected in two cricket cracker samples below the MQL (between 0.4 and 1.4 µg/g), and in the other, a very high amount of this FPC was found (1.9 µg/g in the Ins-C-Oreg). All these novel crackers demonstrated concentrations that exceeded those reported in other papers for traditional cereal-based crackers, between 0.026 and 0.620 μg/g [22,43]. In addition, their AA concentrations were higher than 0.4 μg/g (reference value recommended for all cereal-based products, apart from spice bread [41]). However, these high concentrations are not due to the insect flour used (only 8%) but result from the combination of all other ingredients such as sunflower seeds, golden flax seeds, dried tomato, dried black olives, tomato paste, etc. [42,43,44], together with the processing conditions, which can also affect the presence of the contaminant in the final product.

On the other hand, different amounts of furanic compounds were found in the insect-based samples analyzed (Table 5). HMF and F are commonly studied as MR and sugar pyrolysis intermediates, formed during heating processes of foods. HMF and F are formed by the decomposition of hexoses and pentoses, respectively, and MF can be generated through the degradation of HMF. As can be seen in Table 5, in the Ins-F sample only F was detected. This FPC can be produced during the slaughter of the cricket, since thermal processes, such as cooking or drying in an air oven, can take place during the elaboration of the flour. Besides, the samples with the highest content of cricket flour (and the highest amount of proteins, according to the food label) presented the highest F content (Ins-B-BittCocoa, Ins-F-Choc and Ins-B-Pean), especially those containing chocolate (107 µg/g in Ins-B-BittCocoa and 40 µg/g in Ins-F-Choc). Regarding HMF, samples with a large amount of sugar in their food label, which were prepared with dried fruits (Ins-B-Pine-Coco) or dried fruits and chocolate (Ins-B-Choc-Cher) were those with the highest HMF content. However, compared to the concentrations of HMF found in other cereal bars analyzed in this work (between 333 and 706 µg/g of HMF, depending on the other ingredients included in the formulation), the insect-bars showed a lower concentration of this FPC (between 2.5 and 41 µg/g). This fact can be attributed to their low starch content, because in these insect-based products the cereals (oatmeal flakes, wheat flour, corn, rice, etc.) replaced cricket flour. In addition, some ingredients with an important content of furanic compounds that are usually included at high amounts in common cereal bars, such as glucose and fructose syrups, are added to these products at lower levels or even replaced by other ingredients. Consequently, the levels of HMF, MF and F are reduced in these insect-based foods. These results revealed that insect-bars can be a good alternative to traditional cereals bars. The replacement of cereals with insect flour and the use of other ingredients can help to reduce the presence of furanic compounds in the diet.

## 4. Conclusions

A mesostructured silica functionalized with amino groups has been used for the first time as a sorbent in SPE for the simultaneous extraction and analysis by HPLC-QqQ-MS/MS of HMF, MF, F and AA in insect-based foods. The method was validated, showing good linearity, precision, accuracy, MDL and MQL, and applied to eleven products (bars, crackers and flours). Results reveal that these NF can be a good alternative to traditional foods to reduce dietary exposure to furanic compounds, mainly HMF. However, when designing insect-based food formulas, the control of AA is necessary to ensure healthier NF. The impact of insect-based foods on exposure to FPC needs to be carefully evaluated.

## Figures and Tables

**Table 1:** Mass spectrum parameters for acrylamide (AA), furfural (F), 5-methylfurfural (MF) and hydroxy-methyl-furfural (HMF) using the developed HPLC-QqQ-MS/MS method.

Analyte	Ionization Mode	Precursor Ions *m/z*	MS^2^ Product Ions *m/z* (CE, V)	Dwell Time (s)
AA	ESI (+)	72.0	53.5 (6.5), 54.8 * (5)	0.5
F	ESI (+)	97.0	40.7 (11.5), 69.3 (7), 95.7 * (5)	0.5
MF	ESI (+)	110.9	53.0 (14), 54.9 (12), 81.0 (12), 109.6 *(5)	0.5
HMF	ESI (+)	127.0	53.0 (22), 80.6 (14), 108.8 *(10)	0.5

* Ions used for quantification.

**Table 2:** Textural properties of the prepared materials and functionalization degree of SBA-15-LP-NH_2_.

Material	S_BET_^a^ (m^2^/g)	Pore Volume (cm^3^/g)	Pore Size (Å)	L_0_^b^ (mmol NH_2_ Groups/g)
SBA-15-LP	482	1.7	38 91	-
SBA-15-LP-NH_2_	357	1.3	36 89	0.96

^a^ S_BET_: Specific surface area estimated by Brunauer-Emmett-Teller method. ^b^ L_0_: Functionalization degree. -: not functionalized.

**Table 3:** Linearity, limits and matrix effect of the proposed method in selected insect-based samples.

Analyte ^a^	Sample	Linear Range (µg/mL)	Matrix Matched Calibration (R^2^)	MDL (µg/g) ^b^	MQL (µg/g) ^b^	ME (%) ^c^
AA	Ins-B-Pine-Coco	0.1–10	y = 9.4 × 10^7^*x* + 1.7 × 10^6^ (0.999)	0.4	1.3	112
	Ins-C-Toma	0.1–10	y = 1.1 × 10^8^*x* + 5.6 × 10^6^ (0.998)	0.8	1.4	142
	Ins-F	-	-	-	-	-
F	Ins-B-Pine-Coco	0.5–10	y = 5.7 × 10^6^*x* + 8.5 × 10^5^ (0.998)	2.4	7.9	15
	Ins-C-Toma	0.5–10	y = 5.0 × 10^6^*x* + 6.1 × 10^5^ (0.997)	2.7	8.8	2
	Ins-F	0.5–10	y = 4.7 × 10^6^*x* + 3.3 × 10^6^ (0.999)	2.6	8.5	−6
MF	Ins-B-Pine-Coco	0.2–10	y = 3.3 × 10^7^*x* + 4.0 × 10^6^ (0.996)	0.9	3.1	19
	Ins-C-Toma	0.5–10	y = 2.9 × 10^7^*x* + 1.6 × 10^6^ (0.998)	1.9	6.5	5
	Ins-F	0.5–10	y = 2.8 × 10^7^*x* + 5.0 × 10^6^ (0.995)	1.7	5.7	0
HMF	Ins-B-Pine-Coco	0.1–10	y = 3.6 × 10^7^*x* + 9.2 × 10^6^ (0.991)	0.5	1.5	27
	Ins-C-Toma	0.2–10	y = 1.0 × 10^7^*x* + 4.5 × 10^5^ (0.998)	1.0	3.3	−65
	Ins-F	0.2–10	y = 1.1 × 10^7^*x* + 2.9 × 10^6^ (0.997)	0.6	2.1	−60

^a^ AA: acrylamide; F: furfural; MF: 5-methylfurfural; HMF: hydroxy-methyl-furfural. ^b^ MDL: method detection limit and MQL: method quantification limit. ^c^ ME: matrix effect calculated as: (slope matrix-matched/slope solvent-based) − 1) × 100.

**Table 4:** Precision and accuracy of the proposed method at two concentration levels ^a^.

Analyte	Level ^a^	Recovery (% ± SD) ^b^	Intra-Day Precision (% RSD) ^b^	Inter-Day Precision (% RSD) ^b^
AA	Low	70 ± 6	8	11
	High	70 ± 8	11	10
F	Low	91 ± 10	11	13
	High	83 ± 5	8	14
MF	Low	94 ± 7	7	11
	High	79 ± 6	8	11
HMF	Low	101 ± 3	3	5
	High	96 ± 3	5	8

^a^ Low level: MQL level (1.3 µg/g for AA; 7.9 µg/g for F; 3.1 µg/g for MF and 1.5 µg/g for HMF); high level: 50 µg/g. ^b^ Data obtained using Ins-B-Pine-Coco as representative sample.

**Table 5:** Content of acrylamide (AA), furfural (F), 5-methylfurfural (MF) and hydroxy-methyl-furfural (HMF) in the different insect-based food samples analyzed with the proposed method.

Sample	AA (µg/g ± SD)	F (µg/g ± SD)	MF (µg/g ± SD)	HMF (µg/g ± SD)
Ins-B-Pean	n.d	37 ± 1	4.2 ± 0.3	n.d
Ins-B-BittCocoa	n.d	107 ± 3	17 ± 1	n.d
Ins-B-Pine-Coco	n.d	<MQL	<MQL	15 ± 2
Ins-B-Choc-Ora	n.d	n.d	6.9 ± 0.8	<MQL
Ins-B-Choc-Cher	n.d	9 ± 2	14 ± 4	41 ± 13
Ins-B-Appl	n.d	n.d	<MQL	2.5 ± 0.4
Ins-C-Oreg	1.9 ± 0.3	9 ± 1	13 ± 1	n.d
Ins-C-Toma	<MQL	20 ± 6	6.8 ± 0.6	n.d
Ins-C-Oli	<MQL	11 ± 2	8 ± 1	n.d
Ins-F	n.d	8 ± 1	n.d	n.d
Ins-F-Choc	n.d	40 ± 1	10 ± 1	7 ± 2
Cer-B-Haze	n.d	10 ± 3	<MQL	333 ± 38
Cer-B-Choc-Ora	n.d	n.d	<MQL	402 ± 30
Cer-B-Cran	n.d	n.d	n.d	706 ± 51

n.d = not detected; <MQL: below the limit of quantification of the method.

## Supplementary Materials

The following are available online at https://www.mdpi.com/article/10.3390/foods10071557/s1. Table S1: List of ingredients of analyzed samples in this work; Table S2: Nutritional facts of samples analysed in this work; Table S3: Instrumental validation parameters of the HPLC-QqQ-MS/MS method. Figure S1: (**a**) Recovery percentages (%) of AA, F, MF and HMF in standard solutions (80 µg/mL) using different amounts of sorbent. (**b**) Recovery percentages (%) of AA, F, MF and HMF in standard solutions (10 µg/mL and 5 µg/mL) using 100 mg of sorbent. Figure S2: Recovery percentage (%) of AA, F, MF and HMF obtaining in: (**a**) Optimization of elution with different solvents (3 mL) using standard solutions (5 µg/mL), (**b**) Optimization of the elution volume (2 mL) with the two best extraction solvents. Figure S3: Chromatogram of acrylamide (AA) (*m/z* 72.0 > 54.8), hydroxy-methyl-furfural (HMF) (m/z 127.0 > 108.8), furfural (F) (*m/z* 97.0 > 95.7), 5-methylfurfural (MF) (*m/z* 110.9 > 109.6) and in three representative contaminated samples (Ins-F, Ins-C-Toma and Ins-B-Pine-Coco).
