# Supplementary material for: Simultaneous Determination of Furanic Compounds and Acrylamide in Insect-Based Foods by HPLC-QqQ-MS/MS Employing a Functionalized Mesostructured Silica as Sorbent in Solid-Phase Extraction
Source: Foods. 2021 Jul 5;10(7):1557. doi: 10.3390/foods10071557 (PMC8305596; doi:10.3390/foods10071557)
Supplement: Supplementary file 1 [file foods-10-01557-s001.zip › foods-1267854-supplementary.pdf]

Supplementary Materials

Table S1. List of ingredients of analyzed samples in this work.

Code	Type of food	Description	Ingredients
**Ins-B-Pean**	Insect bar	Peanut Butter & Cinnamon	Peanut butter (22%) (peanuts 100%), cricket flour (*Acheta domesticus*) (20 %), dates, fava bean protein, chicory root fiber, cocoa butter, pumpkin protein, dates, cinnamon (1.7%).
**Ins-B-BittCocoa**	Insect bar	Bitter Cocoa & Sesame	Cricket flour (*Acheta domesticus*) (20 %), sesame protein (19 %), date syrup, tahini (100% sesame seeds), cocoa mass (15%), Fava bean protein, cocoa powder (3 %), chicory root fiber.
**Ins-B-Pine-Coco**	Insect bar	Pineapple & Coconut	Dates, almonds, dried pineapple (16 %), cricket flour (*Acheta domesticus*) (10 %), coconut flakes (8 %), lime juice concentrate, dried apricots, rice flour.
**Ins-B-Choc-Ora**	Insect bar	Dark Chocolate & Orange	Dates, almonds, dark chocolate (cocoa mass, sugar, cocoa butter, vanilla powder) (12 %), cricket flour (*Acheta domesticus*) (10 %), orange juice concentrate (2 %), orange oil (0.4 %).
**Ins-B-Choc-Cher**	Insect bar	Dark Choco & Sour Cherry	Dates, almonds, dried sour cherries (15 %), cricket flour (*Acheta domesticus*) (10 %), dark chocolate (cocoa mass, sugar, cocoa butter, vanilla powder) (9 %), aronia juice concentrate.
**Ins-B-Appl**	Insect bar	Apple, cinnamon and caramel	Dates, whole almonds, caramel (cane sugar, glucose syrup, sweetened condensed milk, butter, Guérande salt) (9%), whole almond puree, honey, whole dehydrated cricket powder (*Acheta domesticus*) (5.5%), dried apple (5%), applesauce (3.5%), cashews, hemp flour, shelled hemp seeds, cinnamon, salt.
**Ins-C-Oreg**	Insect cracker	Oregano and thyme	Sunflower seeds, golden flax seeds, dried tomatoes, cricket flour (*Acheta domesticus*) (8%), onion, spice mix (1.9%), oregano (0.5%), thyme (0.5%), unrefined sea salt.

**Ins-C-Toma**	Insect cracker	Tomato and Basil	Sunflower seeds, golden flax seeds, carrot, tomato paste (10%) (tomatoes 93%, salt 7%), cricket flour (*Acheta domesticus*) (8 %), onion, dried basil (1%), spice mix (0.7%).
**Ins-C-Oli**	Insect cracker	Black Olives	Sunflower seeds, golden flax seeds, dried black olives (11%), dried tomatoes, cricket flour (*Acheta domesticus*) (8%), onion, ground coriander, rosemary, unrefined sea salt.
**Ins-F**	Insect flour	Cricket Flour	House cricket (*Acheta domesticus*) (100 %).
**Ins-F-Choc**	Insect flour	Flour with chocolate	Pea protein, cricket flour (*Acheta domesticus*) (10%), pumpkin protein, chia protein, defatted cocoa powder (5%), dried rice syrup, rice starch, rice flour, sucralose, sunflower lecithin, chocolate aroma (0.5%).
**Cer-B-Haze**	Cereal bar	Hazelnut	Integral oatmeal flakes (32%), glucose and fructose syrup, corn flakes (8%), roasted hazelnuts (8%), extruded wheat and rice (6%), rice flakes (6%), sunflower oil (1%), hazelnut paste, maltodextrin, emulsifier: sunflower lecithin, natural aroma.
**Cer-B-Choc-Ora**	Cereal bar	Chocolate and orange	Wheat flour (18%), glucose and fructose syrup, rolled oatmeal flakes (9%), chocolate chips (8%), corn (7%), sugar, rice (5%), rice flour (3%), sunflower oil, juice orange (1%), lean cocoa powder, fructose syrup, concentrated apple puree, moisturizer, wheat starch, dextrose, salt, wheat malt flour, barley malt flour, emulsifier: sunflower lecithin, malt extract barley, acidulant (E330), natural orange aroma, orange pulp, cocoa butter, gelling agent: pectins, aroma.
**Cer-B-Cran**	Cereal bar	Cranberry	Rolled whole oat flakes (24%), glucose and fructose syrup, glucose syrup, wheat flour (14%), corn (5%), rice (4.5%), lingonberries (3.9%), rice flour (2.9%), sugar, sunflower oil, humectant: glycerin, salt, malted wheat flour, malted barley flour, concentrated apple juice, acidifier: citric acid, natural aroma, emulsifier: sunflower lecithins, barley malt extract.

Table S2. Nutritional facts of samples analyzed in this work.

	Per 100 g						
**Sample**	**Fat (g)**	**of which saturated (g)**	**Carbohydrates (g)**	**of which sugars (g)**	**Fibre (g)**	**Protein (g)**	**Sodium (g)**
Ins-B-Pean	23	8.1	18	14	5.6	34	0.2
Ins-B-BittCocoa	26	10	21	14	6	34	0.3
Ins-B-Pine-Coco	19	6	41	39	9.4	15	0.09
Ins-B-Choc-Ora	23	5.2	37	35	9.3	15	0.09
Ins-B-Choc-Cher	18	4.1	42	39	8.7	15	0.09
Ins-B-Appl	21.1	3.1	42.2	38.2	7.6	13.6	0.28
Ins-C-Oreg	36	4.6	20	5	9.2	22	1.6
Ins-C-Toma	36	4.6	20	5	10	21	3
Ins-C-Oli	34	4.4	20	1	12	21	2
Ins-F	20	5.2	0.5	0	9.5	70	0.8
Ins-F-Choc	5.2	1.7	3.5	0.1	7.7	74	2.8
Cer-B-Haze	13	1.4	65	25	4.7	7.4	0.6
Cer-B-Choc-Ora	6.3	2	74	30	3.3	6.1	0.66
Cer-B-Cran	6.9	2.9	74	26	3.9	5.4	0.46

Table S3. Instrumental validation parameters of the HPLC-QqQ-MS/MS method.

Analyte	Linear range[a] (µg/mL)	Solvent-based standard calibration (R^2)	LOD[b] (µg/mL)	LOQ[c] (µg/mL)	Level[d]	Repeatability (% RSD)	Within-laboratory reproducibility (%RSD)
AA	0.05-10	$y = 4.7x10^7x + 2.1x10^6$	0.03	0.10	Low	4	1
		(0.999)			High	4	6
F	1-10	$y = 5.0x10^6x + 5.2x10^5$	0.22	0.72	Low	4	7
		(0.998)			High	3	9
MF	0.5-10	$y = 2.8x10^7x + 1.9x10^6$	0.05	0.17	Low	4	8
		(0.999)			High	1	5
HMF	0.05-10	$y = 2.9x10^7x + 5.4x10^6$	0.01	0.03	Low	7	4
		(0.996)			High	8	1

[a] Working standard solution (prepared in water-MeOH, 90:10, v/v). [b] Limit of detection (LOD) and [c] limit of quantification (LOQ). [d] Low level: 0.5 mg/L; High level: 2.5 mg/L.

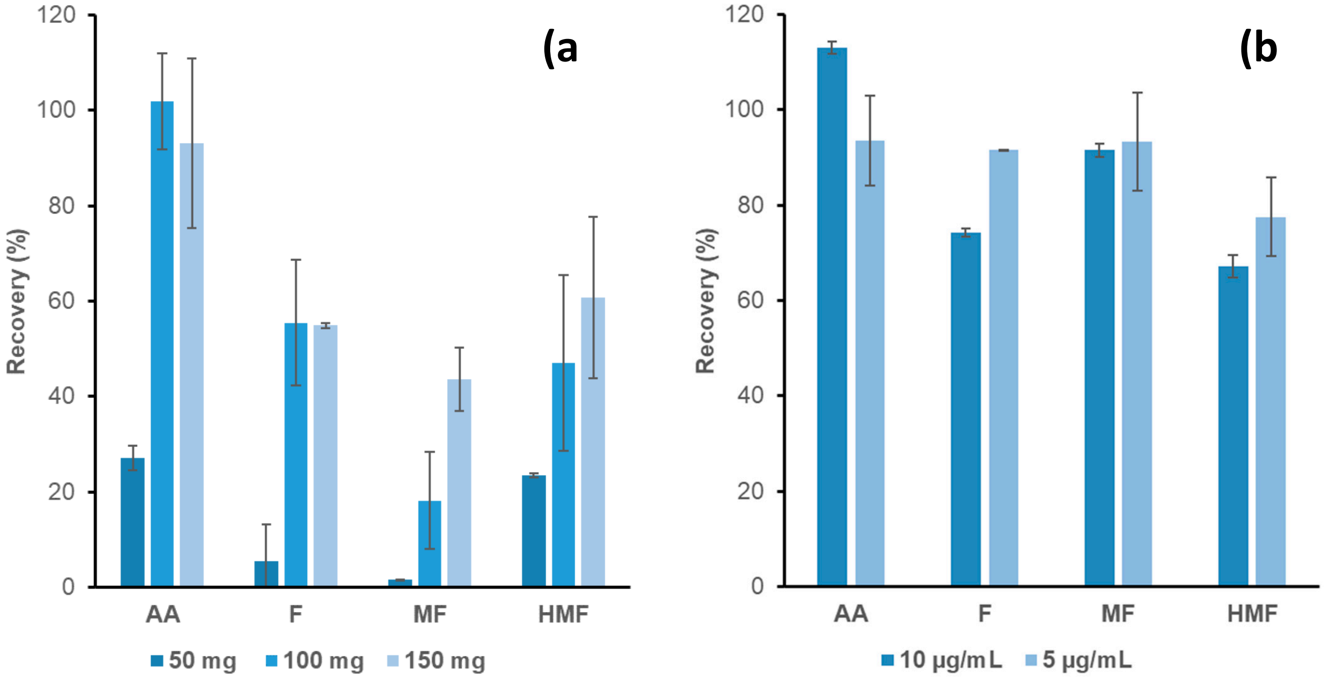

Figure S1. (**a**) Recovery percentages (%) of AA, F, MF and HMF in standard solutions (80 µg/mL) using different amounts of sorbent. (**b**) Recovery percentages (%) of AA, F, MF and HMF in standard solutions (10 µg/mL and 5 µg/mL) using 100 mg of sorbent.

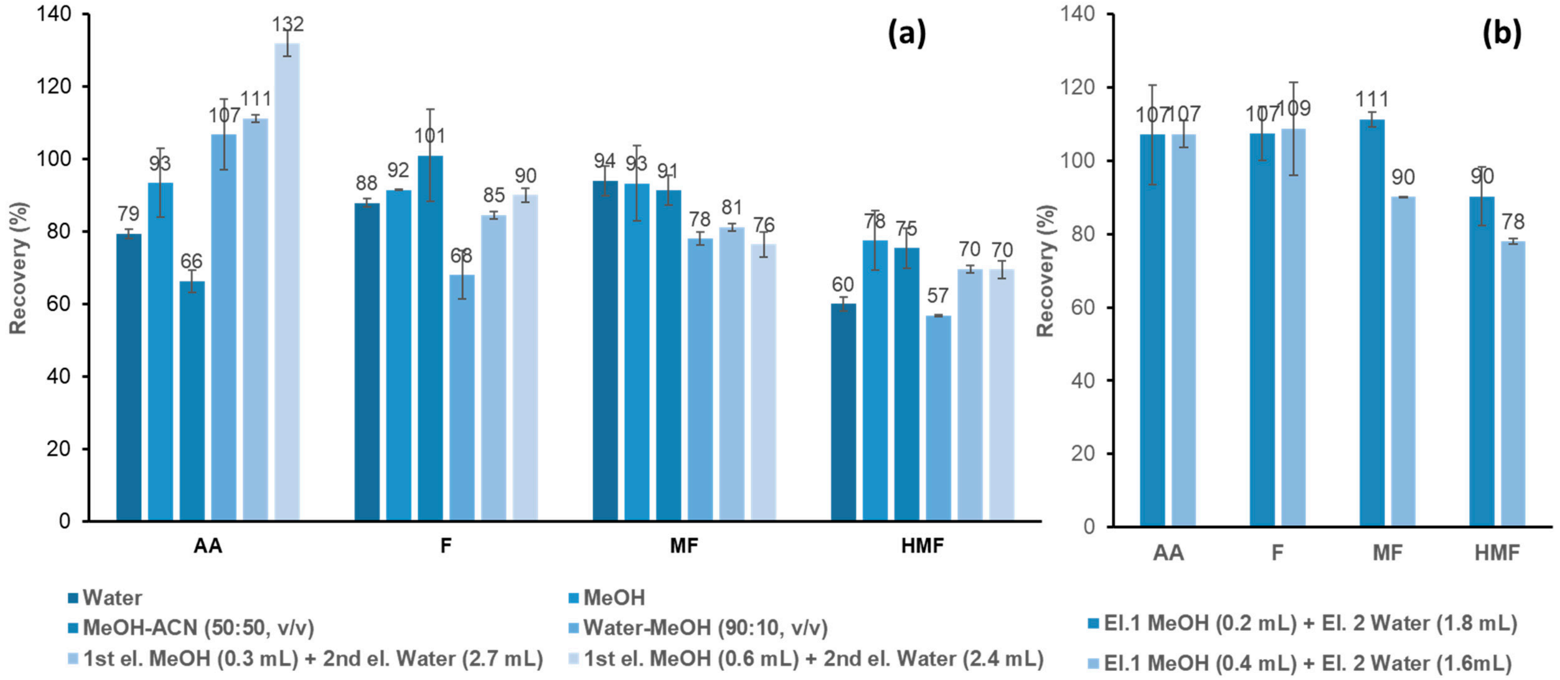

Figure S2. Recovery percentage (%) of AA, F, MF and HMF obtaining in: (**a**) Optimization of elution with different solvents (3 mL) using standard solutions (5 µg/mL), (**b**) Optimization of the elution volume (2 mL) with the two best extraction solvents.

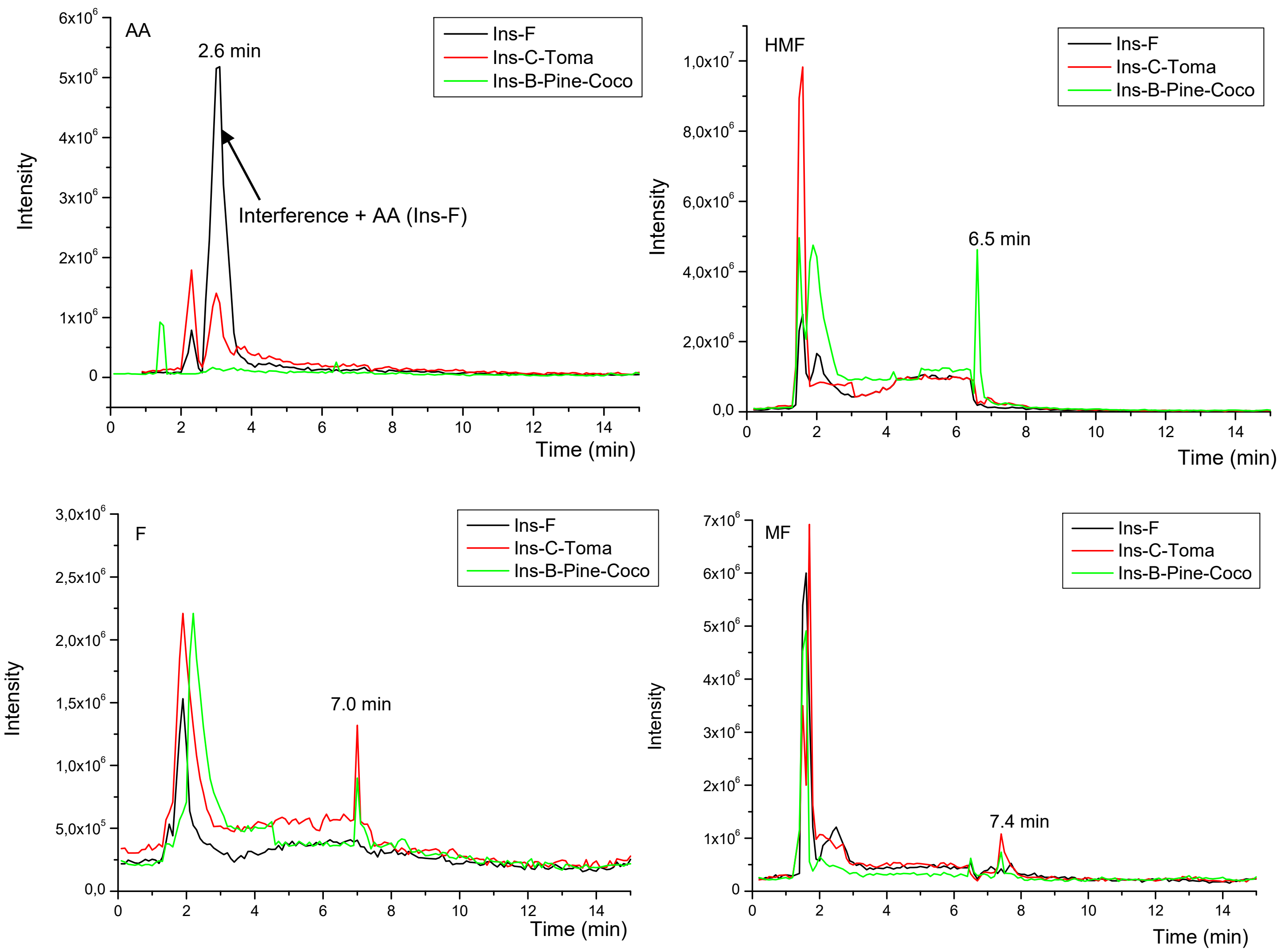

Figure S3. Chromatogram of acrylamide (AA) (m/z 72.0 > 54.8), hydroxymethylfurfural (HMF) (m/z 127.0 > 108.8), furfural (F) (m/z 97.0 > 95.7), 5-methylfurfural (MF) (m/z 110.9 > 109.6) and in three representative contaminated samples (Ins-F, Ins-C-Toma and Ins-B-Pine-Coco).
